# Gold Finger: Metal Jewellery as a Disease Modifying Antirheumatic Therapy!

**DOI:** 10.1155/2009/518976

**Published:** 2009-10-07

**Authors:** T. Hlaing, S. Ramteke, K. Binymin

**Affiliations:** ^1^Rheumatology Department, Southport and Ormskirk Hospital NHS Trust, Liverpool University, PR8 6PN, UK; ^2^Radilogy Department, Southport and Ormskirk Hospital NHS Trust, PR8 6PN, UK

## Abstract

Polyarticular psoriatic arthritis is a chronic, progressive and disabling auto-immune disease often affecting the small joints of the hands in a symmetrical fashion. The disease can progress rapidly causing joint swelling and damaging cartilage and bone around the joints resulting in severe deformities. We report a very unusual case of a 49-year-old woman who presented with polyarticular psoriatic arthritis affecting all proximal interphalangeal (PIP) joints of both hands except the left ring finger PIP joint. On clinical examination there was no evidence of arthritis in the left ring finger PIP joint. We confirmed the paucity of joint damage in the PIP joint of the left ring finger using more modern imaging modalities such as musculoskeletal ultrasound and MRI scan of the small joints of the hands. All other PIP joints in both hands demonstrated advanced degrees of joint damage secondary to chronic psoriatic inflammatory arthritis. We postulated that wearing a gold wedding ring has helped protecting the PIP joint of the left ring finger from the damaging effect of inflammatory arthritis. The possible mechanisms by which metal jewellery (gold ring) confer protection to adjacent joints was discussed.

## 1. Introduction

**Figure 1:**
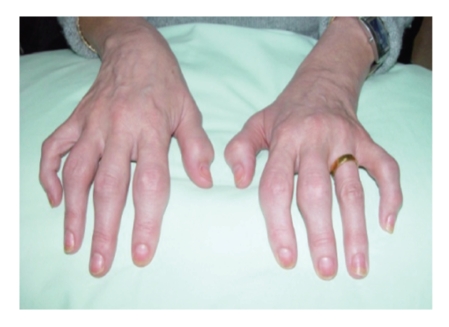
Symmetric polyarticular psoriatic arthritis affecting all the proximal interphalangeal joints of the fingers except the left ring finger PIP joint which seems to be protected by wearing a golden ring. Psoriatic nail changes are also evident in this photo.

**Figure 2:**
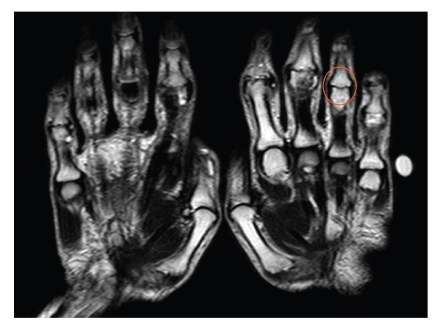
MRI of the proximal interphalangeal joints of both hands confirms sparing of the left ring proximal interphalangeal joint. All other proximal interphalangeal joints show marked synovitis and erosions.

 A 49-year-old right-handed female was admitted for investigation of recent joint swelling affecting the small joints of the hands. She admits to have intermittent arthritis of the hands for the past 3 years. On examination, there was synovitis at the proximal interphalangeal (PIP) joints in a symmetrical distribution of all the fingers of both hands except the PIP joint of the left ring finger (Figure 1). There was also prominent nail (Figure 1) and skin changes consistent with psoriasis. The inflammatory markers were raised, and tests for the RF latex and anticyclic citrullinated peptide antibodies were negative. The diagnosis of symmetric polyarticular psoriatic arthritis was made. Anteroposterior radiograph of the hands showed subchondral erosions with periosteal reaction in all of the PIP joints sparing the left ring finger PIP joint. MRI scan (Figure 2) and ultrasound studies (USs) of the hands confirmed the presence of joint space narrowing and extensive synovitis with articular erosions at the PIPs of all the fingers sparing the left ring finger PIP joint. The patient is married and wore a 22-carat gold wedding ring on the left ring finger throughout her marriage for the last 26 years. 

## 2. Discussion

We observed the sparing of one out of ten identical joints in the hands that are symmetrically involved with psoriatic arthritis. It is difficult to hypothesise that this phenomenon is the result of random sparing of a joint area or natural deselection by a systemic inflammatory process. We believe that the wedding gold ring the patient wore throughout and preceding the onset of the inflammatory arthritis has protected the joint of the left ring finger and prevented the development of florid arthritis. 

 This is the first reported case demonstrating the protective properties of metal rings in psoriatic arthritis. This is also the first time the protective effect of metal ring is demonstrated using more modern imaging techniques such as musculoskeletal MRI and US. We established the paucity of any pathological changes in the ring finger PIP joint and showed that the sparing effect was structural as well as clinical.

The mechanism by which metal jewellery confer protection to adjacent joints is not fully understood and warrants further study. The possible mechanisms that underlie the process of joint protection may include the following.

**(1) Absorption of Gold via Skin**

Seeping of gold locally from the gold ring through to the subcutaneous lymphatic system is a possibility that might explain the sparing of the more proximal MCP joints.

**(2) Mechanical Factor**

It has been noted that joints of congenitally short fingers suffer less erosions in patients with RA [1]. The lower trauma rate from daily use might protect these joints from developing or accelerating the process leading to arthritis. Similarly, metal ring may hinder free use of that finger/joints causing lower level of trauma resulting in less joint damage and erosions.

**(3) Constriction Effect**

An alternative theory is that the constricting effect of a ring might lead to a marginal reduction in local perfusion/pressure of the blood and/or differential drop in the finger temperature. Subsequently, this would mute the expected surge of the various growth factors resulting in lower inflammatory response and synovitis in joints beyond the constricting site.

**(4) Neuronal Factor**

Neuronal factor (sympathetic, neurogenic peptide) could yet be another factor responsible for such phenomena. Patients with stroke and hemiplegia develop less severe disease on the paralysed side (mechanical and neural factors) [2]. Stroke patients would develop skin vasculitis on the nonparalysed side (neural factor) [3]. The metal ring and through stimulation/inhibition of the cutaneous plexus of the skin and deeper nerves in the finger might result in the release of neuropeptide that restrain the inflammatory response.

**(5) Magnetic Effect**

In 1954, Dr. Linus Pauling received a Nobel Prize for his discovery of the magnetic properties of haemoglobin [4]. He found that iron and many electrolytic salts in our blood circulate biomagnetically. Therefore, the proximity of a magnetic field may result in an acceleration of the circulation and the transfer of energy to all areas of our body. In theory one may conclude that local magnetic field generated by metal jewellery could influence blood circulation and enhances biological processes such as acid-base balance and oxidation/reduction reactions resulting in lower inflammatory response in that locality.

## 3. Conclusions

This case strongly supports the idea that wearing metal jewellery could offer benefit to patients with arthritis. We are aware that this phenomenon is not universal for all jewellery wearers. We know that this effect is not limited to one form of arthritis. Similar effect has been reported with rheumatoid arthritis [5] and osteoarthritis. Furthermore, the effect is not unique to gold metals. Copper, silver, and many other metals have been reported to have similar effect to that of gold on arthritis. We hope that this case report would ignite interest in studying this fascinating observation.
